# Ischemic Stroke During Daprodustat Therapy for Renal Anemia: A Report of Three Cases

**DOI:** 10.7759/cureus.57990

**Published:** 2024-04-10

**Authors:** Naohiro Uchio, Shogo Komaki, Akihito Hao, Hideyuki Matsumoto

**Affiliations:** 1 Department of Neurology, Mitsui Memorial Hospital, Tokyo, JPN

**Keywords:** renal anemia, ckd, ischemic stroke, daprodustat, hif-ph inhibitor

## Abstract

Hypoxia-inducible factor prolyl hydroxylase (HIF-PH) inhibitors are increasingly used to treat renal anemia. Ischemic stroke is a rare severe adverse event of HIF-PH inhibitor therapy, and its clinical characteristics have not been described to date. We report three cases of ischemic stroke during treatment with daprodustat, a HIF-PH inhibitor, for anemia associated with non-dialysis-dependent chronic kidney disease (CKD). In two patients, the hemoglobin level exceeded the target hemoglobin level of 13 g/dL for renal anemia. Two patients developed ischemic stroke within two months after the daprodustat administration. None of the three patients experienced a recurrence of ischemic stroke after daprodustat discontinuation. Daprodustat therapy is a risk factor for ischemic stroke, particularly during excessive elevation of hemoglobin levels or the early phases of treatment. Daprodustat should be discontinued to mitigate the risk of ischemic stroke recurrence.

## Introduction

Hypoxia-inducible factor prolyl hydroxylase (HIF-PH) inhibitors are novel oral therapeutic agents for anemia due to chronic kidney disease (CKD). In recent years, six HIF-PH inhibitors, including daprodustat, have been developed and increasingly used for renal anemia [1]. HIF-PH inhibitor therapy can promote endogenous erythropoiesis and has demonstrated efficacy in treating renal anemia. However, amidst its benefits, ischemic stroke has been reported as a rare adverse event [2]. Despite its infrequency, ischemic stroke poses a significant threat to patients undergoing HIF-PH inhibitor therapy, potentially leading to serious outcomes [2]. To date, however, the clinical characteristics of ischemic stroke patients treated with HIF-PH inhibitors have not been well described. Herein, we report three cases of ischemic stroke during treatment with daprodustat for anemia associated with non-dialysis-dependent CKD.

## Case presentation

Case 1

**Figure 1:**
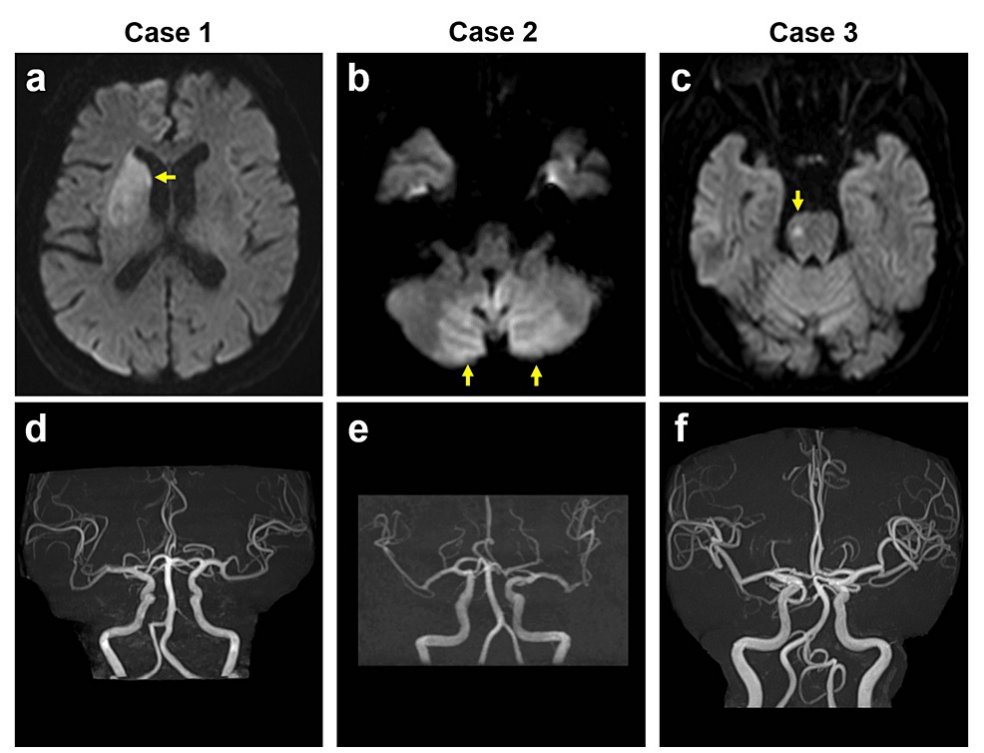
Brain MRI. Axial diffusion-weighted images showing acute infarction in the right basal ganglia due to embolism of the lateral striatum arteries in Case 1 (a, arrow), in both cerebellar hemispheres due to embolism of the posterior inferior cerebellar arteries in Case 2 (b, arrows), and in the right pons due to small-vessel occlusion in Case 3 (c, arrow). MRI angiography showing no occlusions of major cerebral arteries in Case 1 (d), Case 2 (e), or Case 3 (f). MRI, magnetic resonance imaging

The patient was a 79-year-old man who presented with left hemiparesis and who had been taking daprodustat 4 mg for the past month. He had a past history of hypertension. His National Institutes of Health Stroke Scale (NIHSS) score was two. Laboratory test results showed a hemoglobin level of 14.2 g/dL (reference range: 13.7-16.8 g/dL), a creatinine level of 1.45 mg/dL (reference range: 0.65-1.07 mg/dL) (creatinine clearance (Ccr): 37.5 mL/min), and a D-dimer level of 10.4 μg/mL (reference range: 0.0-0.5 μg/mL). Because anemia was well controlled, iron deficiency was not assessed. Brain magnetic resonance imaging (MRI) revealed an acute embolic infarction in the right basal ganglia (Figure 1a; Figure 1d). The sources of embolic infarction were not determined despite the use of Holter electrocardiogram, transthoracic echocardiogram, carotid ultrasound, or deep venous ultrasound of the lower extremities. Hence, daprodustat was discontinued, and edoxaban 30 mg was started. Thereafter, the patient’s symptoms gradually improved. During two years of follow-up, he showed no recurrence of ischemic stroke.

Case 2

The patient was an 85-year-old man who presented with dizziness, dysarthria, and vomiting and who had been taking daprodustat 2 mg for the past five months. He was a current smoker and had a past history of atrial fibrillation, ischemic stroke due to cardioembolism, hypertension, and dyslipidemia. He had been taking apixaban 5 mg, which was discontinued one month prior due to deterioration of renal function. His NIHSS score was three. Laboratory test results showed a hemoglobin level of 13.4 g/dL, a creatinine level of 2.83 mg/dL (Ccr: 17.2 mL/min), and a D-dimer level of 3.6 μg/mL. There was no evidence of iron deficiency (Fe: 67 μg/dL (reference range: 40-188 μg/dL) and ferritin: 100 ng/mL (reference range: 40-465 ng/mL)). Brain MRI revealed an acute infarction in both cerebellar hemispheres due to cardioembolism (Figure 1b; Figure 1e). Daprodustat was discontinued, and edoxaban 15 mg was started. The patient’s symptoms gradually improved. During one year of follow-up, he showed no recurrence of ischemic stroke.

Case 3

The patient was a 74-year-old man who presented with transient dysesthesia in the left lower limb and who had been taking daprodustat 2 mg for the past two months. He had a past history of hypertension, diabetes mellitus, dyslipidemia, and angina pectoris, and had dual antiplatelet therapy with aspirin 100 mg and cilostazol 200 mg. His NIHSS score was zero. Laboratory test results showed a hemoglobin level of 11.4 g/dL, a creatinine level of 3.53 mg/dL (Ccr: 16.7 mL/min), and a D-dimer level of 0.8 μg/mL. Despite anemia, iron deficiency was not assessed. Brain MRI revealed acute infarction due to small-vessel occlusion in the right pons (Figure 1c; Figure 1f). Hence, daprodustat was discontinued. Heparin was added only in the acute phase. During two years of follow-up, the patient showed no recurrence of ischemic stroke.

## Discussion

In this study, we describe three patients who developed ischemic stroke during daprodustat therapy (Table 1). In two patients, the hemoglobin level exceeded the target hemoglobin level of 13 g/dL for renal anemia (Cases 1 and 2). Two patients developed ischemic stroke within two months of daprodustat administration (Cases 2 and 3). One patient developed an ischemic stroke despite dual antiplatelet therapy (Case 3). None of the three patients experienced a recurrence of ischemic stroke, probably because of the discontinuation of daprodustat therapy. Therefore, we speculate that daprodustat therapy plays an important role in the development of ischemic stroke.

**Table 1:** Characteristics of three cases. AF, atrial fibrillation; AP, angina pectoris; Ccr, creatinine clearance; CI, cerebral infarction; Cre, creatinine; CKD, chronic kidney disease; DM, diabetes mellitus; DL, dyslipidemia; Hb, hemoglobin; HT, hypertension; M, male; MRI, magnetic resonance imaging; ND, no data; NIHSS, National Institutes of Health Stroke Scale

	Age/sex	Diseases	Smoking	Daprodustat		Antithrombotic therapy at hospitalization	Ischemic stroke			Laboratory data						Treatment		Outcome	
				Duration (month)	Dose (mg)		Symptoms	NIHSS	MRI findings	Hb (g/dL)	Cre (mg/dL)	Ccr (mL/min)	Fe (μg/dL)	Ferritin (ng/mL)	D-dimer (μg/mL)	Daprodustat discontinuation	Antithrombotic therapy	Follow-up period (year)	Stroke recurrence
1	79/M	CKD, HT	-	1	4	-	Left hemiplegia	2	Embolic infarction in the right basal ganglia	14.2	1.45	37.5	ND	ND	10.4	+	Edoxaban	2	-
2	85/M	CKD, AF, CI, HT, DL	Current smoker	5	2	-	Dizziness, dysarthria, vomitting	3	Embolic infarction in both cerebellar hemispheres	13.4	2.83	17.2	67	100	3.6	+	Edoxaban	1	-
3	74/M	CKD, HT, DM, DL, AP	Past smoker	2	2	Aspirin, Cilostazol	Transient dysesthesia in left lower limb	0	Small-vessel occlusion in the right pons	11.4	3.53	16.7	ND	ND	0.8	+	Heparin	2	-

Ischemic stroke is rare in patients receiving HIF-PH inhibitor therapy [2]. In Japanese phase III trials of daprodustat, ischemic stroke was observed in 0.7% (1/149) of non-dialysis-dependent CKD patients and 0.7% (1/136) of dialysis-dependent CKD patients [3,4]. On the other hand, ischemic stroke was not reported in the other six trials of daprodustat [2]. In all trials, however, patients who experienced recent thromboembolic events were excluded. Therefore, ischemic stroke is expected to be more common in real-world data [2].

The exact mechanism of thromboembolism associated with HIF-PH inhibitor therapy is not fully understood. However, HIF-PH inhibitors are assumed to exert various effects, one of which is erythropoiesis: HIF-PH inhibitors induce the gene transcription of erythropoietin, which can initiate hypoxia-induced responses, resulting in erythropoiesis [5,6]. Thus, thromboembolism is considered to be caused by the erythropoietic effects of HIF-PH inhibitor therapy. Currently, only a few risk factors have been suggested, such as excessive or acute elevation of hemoglobin levels and iron deficiency [2,7].

The CKD guidelines recommend a target hemoglobin level of <13 g/dL [2,7]. Excessive elevation of hemoglobin is believed to increase blood viscosity and increase the risk of thromboembolism. Furthermore, an acute increase in hemoglobin (>0.5 g/dL/week) can also trigger thromboembolism [8]. Because the hemoglobin level typically reaches a steady state within two months after daprodustat administration, patients are at risk for thromboembolism until reaching a steady state [9,10].

HIF-PH inhibitor therapy also affects iron metabolism, which may result in iron deficiency and increase thromboembolic risks [8]. Iron deficiency can lead to thrombocytosis due to the inability to suppress platelet production and can also reduce the deformability of microcytic red blood cells [11,12]. These mechanisms might increase blood viscosity. Unfortunately, in our patients, iron deficiency was not sufficiently evaluated. Therefore, the causal relationship between ischemic stroke under daprodustat therapy and iron deficiency is unknown. At least, however, daprodustat therapy must have played a certain role in the development of ischemic stroke, likely through excessive elevation of hemoglobin levels.

## Conclusions

The development of ischemic stroke represents a critical concern for the management of patients receiving daprodustat therapy for renal anemia. Ischemic stroke may occur during excessive elevation of hemoglobin levels >13 g/dL or during the first two months after daprodustat administration, probably due to the excessive stimulation of erythropoiesis. Thus, when an ischemic stroke occurs, daprodustat should be discontinued to prevent recurrence and ensure patient safety.
